# Síndrome de Takotsubo, Bloqueio Atrioventricular Completo e Parada Cardíaca: Um Desafio Clínico

**DOI:** 10.36660/abc.20250245

**Published:** 2025-12-08

**Authors:** Ana Abrantes, Catarina Gregório, Miguel Raposo, Nelson Cunha, Dulce Brito, Fausto J. Pinto

**Affiliations:** 1 Department of Cardiology Unidade Local de Saúde Santa Maria Lisboa Portugal Department of Cardiology – Unidade Local de Saúde Santa Maria, Lisboa – Portugal; 2 CAML Lisboa Portugal CAML, Lisboa – Portugal; 3 CCUL@RISE Lisboa Portugal CCUL@RISE, Lisboa – Portugal; 4 Faculdade de Medicina Universidade de Lisboa Lisboa Portugal Faculdade de Medicina – Universidade de Lisboa, Lisboa – Portugal

**Keywords:** Bloqueio Atrioventricular Completo, Parada cardíaca, Marca-passo, Síndrome de Takotsubo

## Abstract

O bloqueio atrioventricular completo pode estar associado à síndrome de Takotsubo, e a decisão sobre o implante do dispositivo nesses pacientes é desafiadora. Apresentamos o caso de uma mulher de 76 anos internada após parada cardíaca extra-hospitalar, que foi revertida após tratamento com desfibrilação. O eletrocardiograma pós-ressuscitação documentou bloqueio atrioventricular completo, e o ecocardiograma revelou disfunção ventricular esquerda grave com balonização apical. A angiografia coronária excluiu doença arterial coronariana, e a síndrome de Takotsubo foi diagnosticada com base nas características clínicas e de imagem. O bloqueio atrioventricular persistiu apesar da normalização da função ventricular esquerda; portanto, a parada cardíaca foi considerada secundária a uma arritmia ventricular induzida por bloqueio atrioventricular, e um marca-passo, em vez de um cardioversor-desfibrilador implantável, foi implantado. Durante o acompanhamento, o bloqueio atrioventricular completo persistiu, com 99% da estimulação ventricular observada após a investigação do dispositivo. Este caso corrobora a hipótese de que o estresse do bloqueio atrioventricular pode desencadear a síndrome de Takotsubo, que é agravada pela persistência de anormalidades de condução, apesar da reversão dos defeitos ventriculares. Portanto, a implantação precoce de um dispositivo cardíaco deve ser cuidadosamente considerada caso a caso.

## Introdução

A síndrome de Takotsubo é uma condição cardíaca caracterizada por disfunção ventricular esquerda transitória com movimento anormal da parede, tipicamente balonização apical, que se estende além do território da artéria coronária.^1^ Essa condição afeta predominantemente mulheres na pós-menopausa e geralmente está associada a um estressor emocional ou físico.^1^ No entanto, em até um terço dos pacientes, nenhum gatilho é identificado.^1^ Essa condição está associada à alta morbimortalidade e o tipo de gatilho é um importante fator prognóstico, com maiores taxas de complicações relatadas em pacientes com um estressor físico.^2 , 3^ A fisiopatologia dessa doença ainda é pouco compreendida. Evidências recentes sustentam que o sistema simpático desempenha um papel central com um excesso de catecolaminas que leva à disfunção contrátil regional, bem como à inflamação miocárdica secundária.^1^ As complicações de Takotsubo incluem arritmias com risco de vida, como taquiarritmias ventriculares, relatadas em 3-8% dos pacientes, ou bradiarritmias, com até 2,9% dos pacientes apresentando bloqueio atrioventricular.^2 , 3^ No entanto, arritmias com risco de vida, bem como parada cardíaca, podem ser a apresentação inicial de Takotsubo. Nessas situações, é difícil determinar se essas arritmias são uma consequência ou agem como um gatilho para essa síndrome.^3^ Portanto, a implantação de um dispositivo cardíaco nesse cenário ainda é uma questão de debate, com poucas evidências disponíveis.

Neste artigo, relatamos o caso de um paciente que apresentou parada cardíaca e síndrome de Takotsubo, no qual o bloqueio atrioventricular completo persistiu após a recuperação da disfunção ventricular, sendo implantado um marca-passo.

## Apresentação do caso

**Figura 1:**
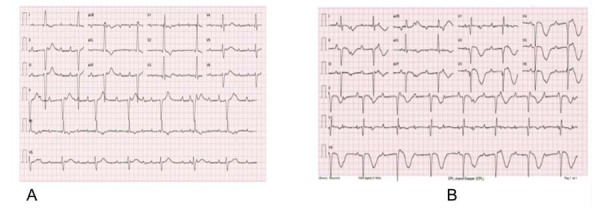
– A) Eletrocardiograma inicial: Ritmo sinusal, bloqueio AV completo, frequência cardíaca de 41 bpm, ritmo de escape com padrão de bloqueio de ramo direito e prolongamento do intervalo QT corrigido (513 ms). B) Eletrocardiograma do 4º dia de internação: Ritmo sinusal, bloqueio AV completo, frequência cardíaca de 44 bpm, inversão da onda T profunda e simétrica e prolongamento do intervalo QT corrigido (599 ms).

**Figura 2:**
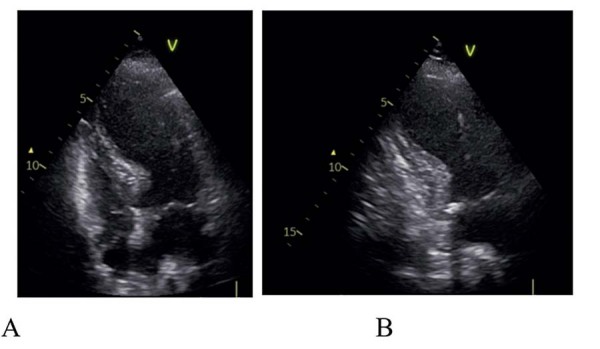

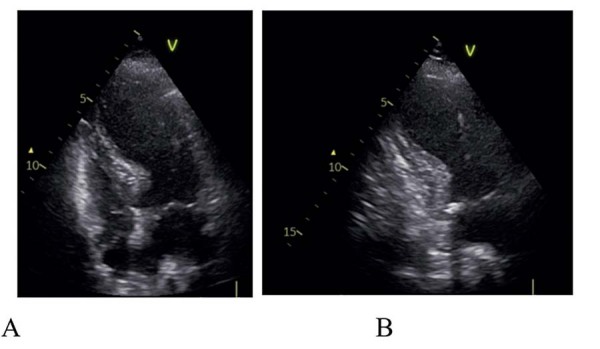
– Ecocardiograma inicial mostrando balonização apical e hipercontratilidade dos segmentos basais em vista de 4 câmaras (A) e vista de 2 câmaras (B).

**Figura 3:**
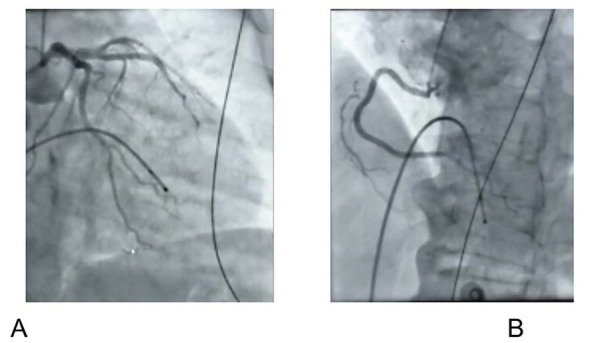
– Angiografia coronária sem doença arterial coronária significativa. Artéria coronária esquerda (A) e artéria coronária direita (B) *.*

Uma mulher de 76 anos foi internada após sofrer parada cardíaca extra-hospitalar enquanto caminhava. A ressuscitação ocorreu após dois ciclos de suporte básico de vida com tratamento com desfibrilador externo automático. Não houve relatos de sintomas prévios e nenhum estressor físico ou emocional foi identificado. O histórico médico da paciente incluía hipertensão e dislipidemia tratadas com indapamida, anlodipina e sinvastatina. Na admissão, ela estava consciente, sem sinais de congestão ou má perfusão. O eletrocardiograma pós-ressuscitação mostrou bloqueio atrioventricular completo com ritmo de escape de 41 bpm com padrão de bloqueio de ramo direito, QRS largo (170 ms) e prolongamento do intervalo QT corrigido (513 ms) ( Figura 1 ). A análise laboratorial mostrou hipocalemia (2,9 mmol/L), troponina elevada (82 ng/L) e elevação acentuada de NTproBNP (4890 pg/mL). O ecocardiograma revelou hipocinesia apical com hipercontratilidade dos segmentos basais e disfunção ventricular esquerda grave (fração de ejeção de 30%) ( Figura 2 ). Um marca-passo transitório foi inserido e a hipocalemia foi corrigida. A angiografia coronária excluiu doença coronária ( Figura 3 ). Durante a hospitalização, ela permaneceu assintomática, com persistência do bloqueio atrioventricular completo e exibiu, no dia 4, um prolongamento acentuado do intervalo QT (599 ms), bem como ondas T profundas invertidas nas derivações precordiais ( Figura 1 ). A ressonância magnética cardíaca foi realizada no dia 14, mostrando hipocinesia e edema no mapeamento T2 dos segmentos apicais, fração de ejeção normal e nenhuma captação de gadolínio, consistente com Síndrome de Takotsubo subaguda. Após a exclusão das causas prevalentes, a hipocalemia foi considerada induzida por indapamida. A paciente permaneceu dependente de um marca-passo externo com bloqueio atrioventricular persistente, apesar da normalização do movimento e da função da parede ventricular esquerda e da normalização da calemia. A parada cardíaca foi considerada secundária a uma taquiarritmia ventricular (possível Torsade de Pointes) induzida por bloqueio atrioventricular (apesar de uma possível contribuição de hipocalemia), e um marca-passo de dupla câmara, em vez de um desfibrilador cardíaco, foi implantado no 18º dia de hospitalização.

Durante um acompanhamento de 24 meses, a paciente permaneceu assintomática, com biomarcadores cardíacos normais e recuperação das anormalidades de movimento da parede. A avaliação do marca-passo mostrou bloqueio atrioventricular completo como ritmo intrínseco, com 30% de estimulação atrial e 99% de estimulação ventricular.

## Discussão

A associação da Síndrome de Takotsubo com bloqueio atrioventricular ou parada cardíaca foi relatada em 2,9% e 5,9% dos pacientes, respectivamente. No entanto, esses números são baseados em pequenos estudos observacionais, e a verdadeira incidência ainda é desconhecida e pode ser menos incomum do que o descrito anteriormente.^2 , 3^ Quando presentes, o bloqueio atrioventricular ou a parada cardíaca são determinantes importantes do prognóstico, associados ao aumento da mortalidade hospitalar.^3^ O mecanismo das arritmias em Takotsubo ainda é desconhecido. Taquiarritmias podem surgir dos efeitos do excesso de catecolaminas, enquanto distúrbios de condução, como bloqueio atrioventricular, podem resultar de um aumento no tônus vagal, bem como refratariedade ventricular anormal (refletida no intervalo QT prolongado) secundária ao edema miocárdico.^4 , 5^ Quando presentes simultaneamente, é difícil determinar se a parada cardíaca ou o bloqueio atrioventricular em si induzem Takotsubo ou se Takotsubo promove um substrato arritmogênico para parada cardíaca ou bloqueio atrioventricular. Dada a natureza reversível do Takotsubo, a implantação de um dispositivo cardíaco na fase aguda é controversa.

Conforme destacado neste caso, pacientes apresentando bloqueio atrioventricular e Takotsubo são geralmente idosos, com substrato para anormalidades degenerativas de condução, com idade média de 69 anos relatada por Jesel et al.^6^ Além disso, o bloqueio atrioventricular tende a persistir apesar da recuperação completa da disfunção ventricular. Em uma coorte de 6 pacientes apresentando Takotsubo e bloqueio atrioventricular que receberam um marca-passo, uma alta porcentagem de estimulação ventricular foi relatada durante um acompanhamento médio de 42 meses, apesar da reversão das anormalidades ventriculares.^6^ Um estudo retrospectivo relatou 8 pacientes com bloqueio atrioventricular completo durante a fase aguda de Takotsubo, 6 deles receberam um marca-passo com bloqueio atrioventricular alto contínuo durante o acompanhamento, e um dos pacientes que não recebeu um dispositivo cardíaco apresentou morte súbita 4 meses após a alta.^7^ No entanto, em alguns casos, bloqueio atrioventricular transitório associado a Takotsubo, onde um marca-passo não é implantado, foi relatado, particularmente em pacientes mais jovens.^8^

No caso apresentado, o bloqueio atrioventricular foi considerado o evento primordial que atuou como estressor físico que levou ao Takotsubo. Essa suposição é corroborada pela idade avançada da nossa paciente, pela persistência do bloqueio atrioventricular durante um acompanhamento de 24 meses, apesar da recuperação da função ventricular esquerda, pela ocorrência tardia de achados eletrocardiográficos tipicamente associados à Takotsubo e pela ausência de um estressor emocional.

## Conclusão

Em conclusão, este caso destaca a importância de considerar o bloqueio atrioventricular como um estressor físico desencadeante da Takotsubo, especialmente em pacientes idosos e quando as anormalidades de condução persistem apesar da reversão dos defeitos ventriculares. Neste relato de caso, a idade do paciente foi um fator importante na decisão de implante de marca-passo, visto que está associada a maiores taxas de doenças do sistema de condução.
