# Supplementary material for: Síndrome de Takotsubo, Bloqueio Atrioventricular Completo e Parada Cardíaca: Um Desafio Clínico
Source: Arq Bras Cardiol. 2025 Dec 8;122(11):e20250245. [Article in Portuguese] doi: 10.36660/abc.20250245 (PMC12711216; doi:10.36660/abc.20250245)
Supplement: Translation

# Translation: Takotsubo Syndrome, Complete Heart Block, and Cardiac Arrest: A Clinical Challenge

**DOI:** 10.36660/abc.20250245i

**Authors:** Ana Abrantes, Catarina Gregório, Miguel Raposo, Nelson Cunha, Dulce Brito, Fausto J. Pinto

**Affiliations:** 1 Portugal Department of Cardiology – Unidade Local de Saúde Santa Maria, Lisboa – Portugal; 2 Portugal CAML, Lisboa – Portugal; 3 Portugal CCUL@RISE, Lisboa – Portugal; 4 Portugal Faculdade de Medicina – Universidade de Lisboa, Lisboa – Portugal

**Keywords:** Complete Atrioventricular Block, Cardiac Arrest, Pacemaker, Takotsubo Syndrome

## Abstract

Complete atrioventricular block may be associated with Takotsubo syndrome, and the decision of device implantation in such patients is challenging. We present a case of a 76-year-old woman admitted after out-of-hospital cardiac arrest that was reverted after defibrillation treatment. Post-resuscitation electrocardiogram documented complete atrioventricular block, and echocardiogram revealed severe left ventricular dysfunction with apical ballooning. Coronary angiography excluded coronary artery disease, and Takotsubo syndrome was diagnosed based on clinical and imaging features. Atrioventricular block persisted despite normalization of left ventricular function; therefore, cardiac arrest was considered secondary to a ventricular arrhythmia induced by atrioventricular block, and a pacemaker, rather than an implantable cardioverter-defibrillator, was implanted. During follow-up, complete atrioventricular block persisted, with 99% of ventricular pacing observed upon device interrogation. This case supports that the stress of atrioventricular block may trigger Takotsubo syndrome, which is reinforced by the persistence of conduction abnormalities despite reversal of ventricular defects. Therefore, early implantation of a cardiac device should be carefully considered on a case-by-case basis.

## Introduction

Takotsubo syndrome is a cardiac condition characterized by a transient left ventricular dysfunction with abnormal wall motion, typically apical ballooning, that extends beyond a coronary artery territory.^1^This condition affects predominantly post-menopausal women and is usually associated with an emotional or physical stressor.^1^However, in up to one-third of patients, no trigger is identified.^1^This condition is associated with high morbimortality and the type of trigger is an important prognostic factor, with higher complication rates reported in patients with a physical stressor.^2 , 3^The pathophysiology of this disease is still poorly understood. Recent evidence supports that the sympathetic system plays a central role with an excess of catecholamines that leads to regional contractile dysfunction, as well as secondary myocardial inflammation.^1^Takotsubo complications include life-threatening arrhythmias such as ventricular tachyarrhythmias, reported in 3-8% of patients, or bradyarrythmias, with up to 2.9% of patients presenting with atrioventricular block.^2 , 3^However, life-threatening arrhythmias, as well as cardiac arrest, may be the initial presentation of Takotsubo. In these situations, it is difficult to determine whether these arrhythmias are a consequence or act as a trigger for this syndrome.^3^Therefore, the implantation of a cardiac device in this setting is still a matter of debate, with scarce evidence available.

In this paper, we report a case of a patient who presented with cardiac arrest and Takotsubo syndrome, in which complete atrioventricular block persisted after ventricular dysfunction recovery, and a pacemaker was implanted.

## Case presentation

A 76-year-old woman was admitted after experiencing out-of-hospital cardiac arrest while walking. Resuscitation occurred after two cycles of basic life support with automated external defibrillator treatment. There were no prior symptoms reported, and no physical or emotional stressors were identified. Patient’s medical history included hypertension and dyslipidemia treated with indapamide, amlodipine, and simvastatin. On admission, she was conscious, with no signs of congestion or malperfusion. Post-resuscitation electrocardiogram showed complete atrioventricular block with an escape rhythm of 41 bpm with a right bundle branch block pattern wide QRS (170ms), and corrected QT interval prolongation (513ms) ( Figure 1 ). Laboratory analysis showed hypokalemia (2.9 mmol/L), elevated troponin (82 ng/L), and marked elevation of NTproBNP (4890 pg/mL). Echocardiogram revealed apical hypokinesis with hypercontractility of basal segments and severe left ventricular dysfunction (ejection fraction of 30%) ( Figure 2 ). A transitory pacemaker was inserted, and hypokalemia was corrected. Coronary angiography excluded coronary disease ( Figure 3 ). During hospitalization, she remained asymptomatic, with persistence of complete atrioventricular block and exhibited, on day 4, a marked prolongation of QT interval (599 ms) as well as deep inverted T waves in precordial leads ( Figure 1 ). Cardiac magnetic resonance was performed on day 14, showing hypokinesia and oedema in T2 mapping of apical segments, normal ejection fraction, and no gadolinium uptake, consistent with subacute Takotsubo Syndrome. After exclusion of prevalent causes, hypokalemia was considered to be induced by indapamide. The patient remained dependent on an external pacemaker with persistent atrioventricular block despite normalization of left ventricular wall motion and function, and normalization of kalemia. Cardiac arrest was considered to be secondary to a ventricular tachyarrhythmia (possible Torsade de Pointes) induced by atrioventricular block (despite a possible contribution of hypokalemia), and a dual chamber pacemaker, rather than a cardiac defibrillator, was implanted on the 18th day of hospitalization.

**Figure 1:** – A) Initial electrocardiogram: Sinus rhythm, complete AV block, heart rate 41 bpm, escape rhythm with a right bundle branch block pattern, and corrected QT interval prolongation (513ms). B) Electrocardiogram of the 4th day of admission: Sinus rhythm, complete AV block, heart rate 44 bpm, deep and symmetrical T wave inversion, and corrected QT interval prolongation (599 ms).

**Figure 2:** – Initial echocardiogram showing apical ballooning and hypercontractility of the basal segments in 4 chamber view (A) and 2 chamber view (B).

**Figure 3:** – Coronary angiography with no significant coronary artery disease. Left coronary artery (A) and right coronary artery (B).

During a follow-up of 24 months, the patient remained asymptomatic with normal cardiac biomarkers and recovery of wall motion abnormalities. Pacemaker interrogation showed complete atrioventricular block as the intrinsic rhythm, with 30% of atrial and 99% of ventricular pacing.

## Discussion

The association of Takotsubo Syndrome with atrioventricular block or cardiac arrest has been reported in 2.9% and 5.9% of patients, respectively. However, these numbers are based on small observational studies, and the true incidence is still unknown and may be less uncommon than previously described.^2 , 3^When present, atrioventricular block or cardiac arrest are important determinants of prognosis, associated with increased in-hospital mortality.^3^The mechanism of arrhythmias in Takotsubo is still unknown. Tachyarrythmias can arise from the effects of catecholamine excess, while conduction disturbances such as atrioventricular block may result from an increase in vagal tone, as well as abnormal ventricular refractoriness (reflected in prolonged QT interval) secondary to myocardial oedema.^4 , 5^When present simultaneously, it is difficult to determine whether cardiac arrest or atrioventricular block itself induces Takotsubo or if Takotsubo promotes an arrhythmogenic substrate for cardiac arrest or atrioventricular block. Given the reversible nature of Takotsubo, the implantation of a cardiac device in the acute phase is controversial.

As highlighted in this case, patients presenting with atrioventricular block and Takotsubo are usually old, with substrate for degenerative conduction abnormalities, with a mean age of 69 years reported by Jesel et al.^6^Furthermore, atrioventricular block tends to persist despite complete recovery of ventricular dysfunction. In a cohort of 6 patients presenting with Takotsubo and atrioventricular block that received a pacemaker, a high percentage of ventricular pacing was reported during a mean follow-up of 42 months, despite reversal of ventricular abnormalities.^6^A retrospective study reported 8 patients with complete atrioventricular block during the acute phase of Takotsubo, 6 of them received a pacemaker with ongoing high atrioventricular block during follow-up, and one of the patients who didn’t receive a cardiac device experienced sudden death 4 months after discharge.^7^Nevertheless, in a few cases, transient atrioventricular block associated with Takotsubo, where a pacemaker is not implanted, has been reported, particularly in younger patients.^8^

In the presented case, atrioventricular block was considered to be the primordial event that acted as a physical stressor that led to Takotsubo. The older age of our patient supports this assumption, persistence of atrioventricular block during a follow-up of 24 months despite recovery of left ventricular function, late occurrence of electrocardiogram findings typically associated with Takotsubo, and absence of an emotional stressor.

## Conclusion

In conclusion, this case highlights the importance of considering atrioventricular block as a physical stressor triggering Takotsubo, especially in older patients and when conduction abnormalities persist despite reversal of ventricular defects. In this case report, the age of the patient was an important feature for the decision of a pacemaker implantation since its association with higher rates of conduction system disease.
